# Highly potent multivalent VHH antibodies against Chikungunya isolated from an alpaca naïve phage display library

**DOI:** 10.1186/s12951-022-01417-6

**Published:** 2022-05-14

**Authors:** Qianlin Li, Fuqiang Zhang, Yi Lu, Huan Hu, Jin Wang, Cheng Guo, Qiang Deng, Conghui Liao, Qin Wu, Tingsong Hu, Zeliang Chen, Jiahai Lu

**Affiliations:** 1 One Health Center of Excellence for Research and Training, School of Public Health, Sun Yat-Sen University, Guangzhou, 510080 China; 2 NMPA Key Laboratory for Quality Monitoring and Evaluation of Vaccines and Biological Products, Guangzhou, 510080 China; 3 Key Laboratory of Tropical Diseases Control, Sun Yat-Sen University, Ministry of Education, Guangzhou, 510080 China; 4 Center for Disease Control and Prevention of Southern Theater Command, Guangzhou, 510060 People’s Republic of China; 5 Health Effects Institute, Boston, 02169 USA; 6 Center for Infection and Immunity, Mailman School of Public Health, Columbia University, New York, NY USA

**Keywords:** Chikungunya, VHH antibody, Naïve phage display library, E2 glycoprotein, Epitope

## Abstract

**Background:**

Chikungunya virus (CHIKV) is a re-emerged mosquito-borne alphavirus that can cause musculoskeletal diseases, imposing a substantial threat to public health globally. High-affinity antibodies are need for diagnosis and treatment of CHIKV infections. As a potential diagnostic and therapeutic agent, the multivalent VHH antibodies is a promising tookit in nanomedicine. Here, we developed potent multivalent VHH antibodies from an alpaca naïve phage display library targeting the E2 glycoprotein of the CHIKV virus.

**Results:**

In the present study, we generated 20 VHH antibodies using a naïve phage display library for binders to the CHIKV E2 glycoprotein. Of these, multivalent VHH antibodies Nb-2E8 and Nb-3C5 had specific high-affinity binding to E2 protein within the nanomolar range. The equilibrium dissociation constant (KD) was between 2.59–20.7 nM, which was 100-fold stronger than the monovalent antibodies’ affinity. Moreover, epitope mapping showed that Nb-2E8 and Nb-3C5 recognized different linear epitopes located on the E2 glycoprotein domain C and A, respectively. A facile protocol of sandwich ELISA was established using BiNb-2E8 as a capture antibody and HRP-conjugated BiNb-3C5 as a detection antibody. A good linear correlation was achieved between the *OD*_*450*_ value and the E2 protein concentration in the 5–1000 ng/mL range (*r* = 0.9864, *P* < 0.0001), indicating its potential for quantitative detection of the E2 protein.

**Conclusions:**

Compared to monovalent antibodies, multivalent VHH antibodies Nb-2E8 and Nb-3C5 showed high affinity and are potential candidates for diagnostic applications to better detect CHIKV virions in sera.

**Graphical Abstract:**

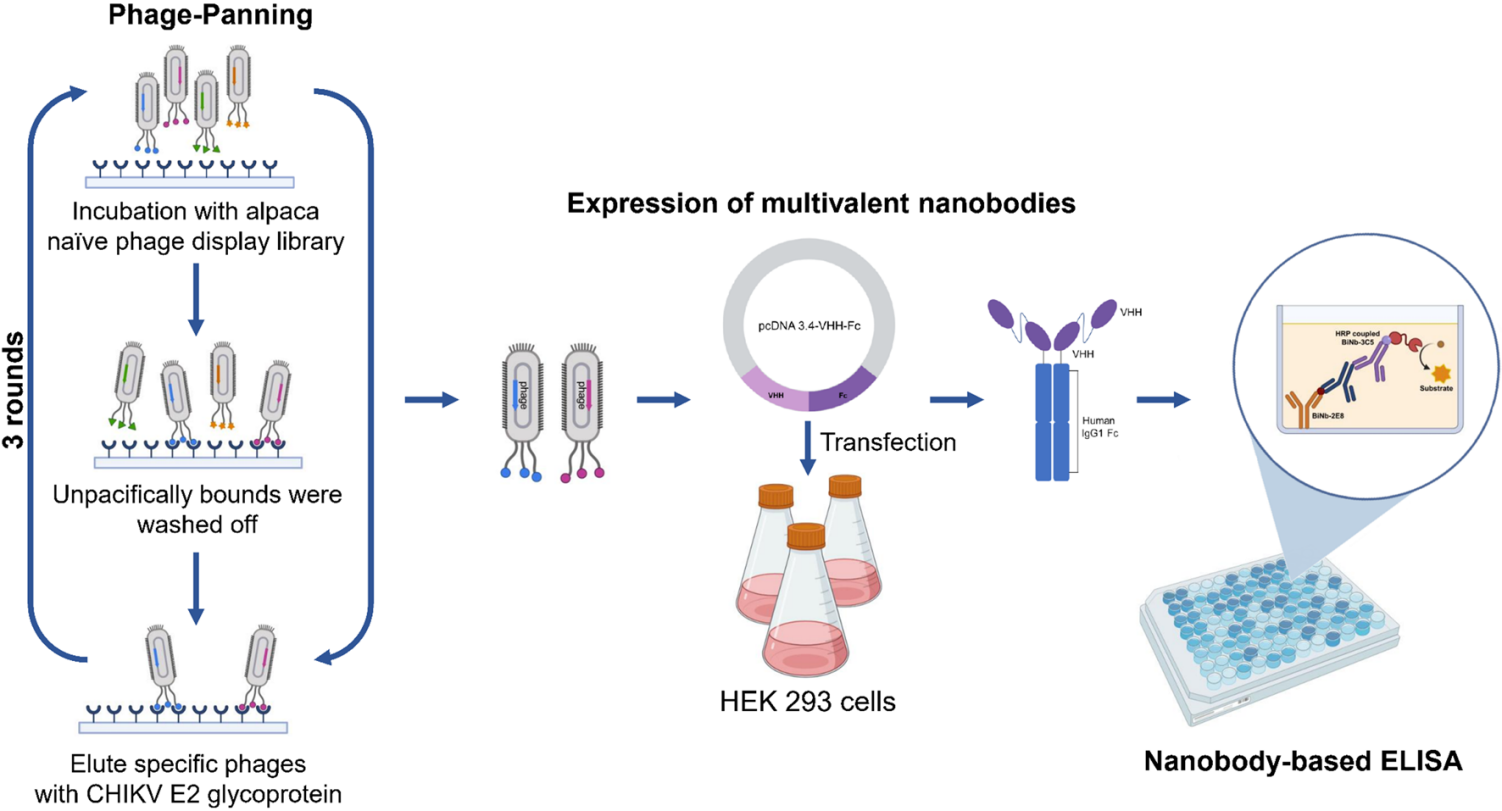

**Supplementary Information:**

The online version contains supplementary material available at 10.1186/s12951-022-01417-6.

## Background

Chikungunya virus (CHIKV) is the causative agent of chikungunya fever (CHIKF), generally causing febrile, arthralgia/arthritis, and rash. The virus is mainly transmitted by the *Aedes aegypti* and *Aedes albopictus* mosquitoes. Human CHIKV infection was first reported in Tanzania in 1953 when the pathogen caused the first major outbreak [1]. In 2007, the *World* *Health* *Organization* (WHO) first described CHIKF “A re-emerging disease in Asia” as a call to increase attention to CHIKV worldwide [2]. In the past decades, CHIKF is prevalent in the Pacific region and Latin America with millions of cases reported over 100 countries [3–5]. A recent outbreak occurred at the China-Myanmar border in 2019, which was imported from Myanmar to Ruili City, Yunnan Province, China. Being highly infectious, it is considered one of the fastest spreading mosquito-borne diseases following malaria and dengue. As there are no licensed antiviral treatments or vaccines available for the CHIKV infection, early diagnosis is critical. While the mortality rate is low for CHIKV infections, they are likely to be misdiagnosed.

CHIKV is an enveloped single-stranded RNA *Alphavirus* [6]. The mature infectious CHIKV is a ~ 70 nm icosahedral enveloped particle in diameter, composed of transmembrane glycoproteins E1, E2, and E3. E1 and E2 glycoproteins assemble into heterodimers with 80 trimeric spikes on the surface of the virions [7, 8]. E1 is a type II transmembrane glycoprotein that mediates the fusion of virus and the host cell membrane [9, 10]. E3 is responsible for the folding of the E2-E1 heterodimer but is cleaved during post-translational maturation [11]. E2 is composed of three immunoglobulin (Ig)-like domains located at the center of an N-terminal domain A, the lateral tip of domain B, and at the lateral tip of a C-terminal domain C. Domain A and domain B are considered to be the binding sites of the Mxra8 receptor on the target cell [12]. Because of this structural advantage, CHIKV glycoprotein E2 appears to be immunodominant with multiple identified epitopes which induce neutralizing antibodies and therefore has a higher serodiagnostic potential.

Nanobodies are single variable domains on heavy chain (VHH) antibodies of molecular weight ~ 15 kDa that are derived from camelid and cartilaginous heavy-chain only antibodies [13, 14]. As the smallest functional antibody, it is widely studied both in *vitro* and in *vivo*. Compared to conventional antibodies, VHHs are highly stable and soluble, easily purified from *E. coli*, and typically have nanomolar binding affinities [15–17]. Specifically, the complementarity-determining region 3 (CDR3) of nanobodies play a key role in recognizing cavities or hidden epitopes such as the virus-binding site of a cell surface receptor [18] and the active site of an enzyme [19, 20]. Screening of VHH antibodies from the naïve library is the superior option for fast diagnosis and efficient treatment. For example, Dong, et al. [21]and Huo, et al. [22] identified the high-affinity nanobodies against SARS-CoV-2 from naïve llama VHH libraries that bind to the S/RBD protein and block its interaction with ACE2. Similarly, Yan, et al. [23] recognized two human procalcitonin (PCT) nanobodies from the naïve phage display library and successfully applied them to develop a sandwich enzyme-linked immunosorbent assay (ELISA), which showed a linear range of PCT between 10–1000 ng/mL.

The current gold standard of CHIKV diagnosis is using virus isolation or virus genome detection assays. Compared to cell culture, fluorescence quantitative PCR (qRT-PCR) analysis is a viable alternative method for viral nucleic acid testing. However, qRT-PCR is problematic due to complicated nucleic acid extraction, which requires highly demanding experimental conditions, is time-consuming, and has an extremely high miss-detection rate [24]. Detection of virions using antibody-based ELISA is a simple and effective approach for identifying and quantitating antigens. To the best of our knowledge, commercial ELISAs are only available for detecting the antibodies for CHIKV (i.e.,  IgM and IgG). However, commercial rapid ELISA tests for detecting CHIKV antigens are not available. Continued trade and international travel growth may also increase the probability of the emergence of vector viruses. Screening individuals with fever for CHIKV infections using rapid diagnostic tests at airports or customs are warranted. This practice can reduce the risk of importing infections to mainland China.

Here, we generated 20 VHH antibodies using a naïve phage display library for binders to the CHIKV E2 glycoprotein. Considering the advantages of activity and affinity, Nb-2E8 and Nb-3C5 were selected as the focus of the present study. Our results suggested that both antibodies could bind to CHIKV E2 protein and virus particles as well as recognize different epitopes. In addition, a 100-fold increase in affinity for the E2 glycoprotein was observed when two VHH antibodies were constructed into multivalent formats. This result can be used for diagnostic applications to detect sera binding proteins and/or virions, either as single VHHs or in combination.

## Materials and methods

### Cell lines and viruses

Vero cells were obtained from the American Type Culture Collection (ATCC) and cultured in Dulbecco Modified Eagle Medium (DMEM) supplemented with 10% fetal bovine serum (FBS) (Thermo Fisher Scientific) and 1% penicillin and streptomycin at 5% CO_2_, 37 ℃. Chikungunya/human/China/GD134/2010 (GenBank Accession: HQ846359) was isolated from the serum of an infected female patient at Guangdong Provincial Center for Disease Control and Prevention. Zika/human/China/GD01/2016 (GenBank Accession: KU740184) was isolated from Guangdong inbound passengers. All experiments involving the CHIKV and ZIKA authentic virus were conducted in biosafety level 2 or higher laboratories. Virus virions were inactivated using β-propiolactone (Sigma-Aldrich) with a concentration of 1:2000 at 4 ℃ for 24 h. Complete inactivation of the virus was confirmed by the lack of replication in a Vero infection experiment. Supernatants containing virus particles were concentrated using PEG-it (SBI Biosciences) overnight at 4 °C and resuspended in phosphate-buffered solution (PBS, pH7.4) for further use.

### VHH antibodies screening from naïve phage display library

A VHH phage display library (AlpaLife) of ~ 2 × 10^9^ cfu was generated using the phagemid vector pADL-10b. *Escherichia coli* (*E.coli*) SS320 cells containing the VHH library were infected with helper phage M13KO7 to produce phages displaying the encoded VHHs as fusion proteins. Biopanning of phages displaying VHHs specific to the Chikungunya/SL-CK1 E2 (Sino Biological) glycoprotein was performed as described previously [25, 26]. After three rounds of panning, 384 individual clones were picked to inoculate 2 × YT (yeast extract tryptone) medium supplemented with 100 μg/mL ampicillin overnight at 37 °C. The cell-free phage supernatant was detected by phage-ELISA with HRP-conjugated goat anti-M13 IgG antibody (Sino Biological). *OD*_*450*_ values ≥ 0.5 and *P* (positive *OD*_*450*_)/*N* (negative *OD*_*450*_) greater than 3 *P/N* ratios were determined as positive clones. Positive candidates were sequenced (Sangon Biotech) and aligned with CDRs amino acid sequence. A five percent bovine serum albumin (BSA) was used as a negative control for each round.

### Expression and purification of VHHs in *E.coli*

For monovalent VHH antibodies, sequences were synthesized (Generay Biotech) and subcloned into pET-SUMO with a tandem N-terminal His-tag, SUMO-tag. Recombinant plasmids were subsequently transformed into *E. coli* BL21(DE3) cells. Protein expression was carried out overnight at 18 °C after 0.5 mM isopropyl β-D-1-thiogalactopyranoside (IPTG) induction at *OD*_*600*_ 0.6. The His-tagged protein was purified by Talon Metal Affinity Resin (Clontech) according to the manufacturer. The eluates were concentrated using a 1.5 mL Microsep advance centrifugal device (PALL) with a molecular weight cut-off of 3 kDa. Purity quality was analyzed by Coomassie-stained sodium dodecyl sulfate–polyacrylamide gel electrophoresis (SDS-PAGE).

### Expression and purification of Fc conjugated multivalent VHH antibodies in HEK293 cells

Bivalent and trivalent VHHs were fused to the Fc region of human IgG1 and cloned into the pcDNA 3.4. Multivalent VHH units were connected through (GGGGS) × 3 flexible linkers. The Fc-fusion constructs were expressed in HEK293 cells at 8% CO_2_ at 37 °C for 1 week. Antibodies in the supernatant were purified using protein A. Purity of all samples was analyzed by size-exclusion high-performance liquid chromatography (SEC-HPLC).

### TEM (Transmission Electron Microscopy) and NTA (Nanoparticle Tracking Analysis) measurements of purified VHHs

The size and structure of VHHs were analyzed by negative stain TEM and NTA. 10 µL of an aqueous solution of samples were applied to copper mesh grids (400 mesh). After deposition for 5 min, the excessive solution was removed with filter paper. Drops of 3% phosphotungstic acid stained on the grid for three minutes and observed on a TEM (JEM-1200EX, JEOL, Tokyo, Japan). All samples were diluted in PBS before measurement on a NanoSight NS300 instrument (Malvern, Worchestershire, UK). Data acquisition and processing were performed using NTA software 3.3. Each sample was recorded three times in 30 s, with detection threshold 3.

### Indirect ELISA to quantitate initial binding

Microtiter plates (Corning) were coated with 2 µg/mL CHIKV E2, 10 µg/mL purified virus virions or the control protein SUMO in a carbonate buffer (CBS, pH9.6) overnight at 4 °C, and blocked with 3% BSA(Sigma-Aldrich) PBS (pH7.4) at 37 ℃. Serial tenfold dilutions of the SUMO-tagged VHHs in 3% BSA were incubated with the immobilized antigen, followed by incubation with HRP-conjugated goat anti-SMT3 (1:2000, CUSABIO). After wash, 100μL of TMB (3,3′,5,5′-tetramethylbenzidine) substrate (TIANGEN) was added to the wells, and reactions were stopped with 100μL of 1 M HCl. Absorbance was measured at 450 nm on an Epoch™ microplate reader (BioTek Instruments Inc., Winooski, VT, USA). The EDIII protein of ZIKA was used as the negative control.

### VHH antibodies were validated by western blotting

Purified CHIKV E2 proteins (20 μg) and CHIKV virions (50 μg) were loaded onto 12% SDS-PAGE and electro-transferred to a polyvinylidene fluoride (PVDF) membrane (Millipore). Following blocking with 5% BSA in 1 × TBST (Tris-buffered saline, 0.05%Tween-20), the membranes were incubated overnight with 1:500 dilution of antibodies. Membranes were probed with an SMT3-HRP conjugated antibody (1:1000, CUSABIO), and revealed with the SuperSignal West Dura ECL reagent (Thermo Fisher Scientific). Chemiluminescence images were visualized with the FluorChem E scanner system (ProteinSimpleSan, Jose, CA, USA).

### Localized Surface Plasmon Resonance (LSPR) assay

LSPR measurements were performed using an OpenSPRTM instrument (Nicoyalife) to determine the affinity of monovalent VHH antibodies to the E2 protein. The COOH chip (Nicoyalife, Canada) was loaded onto the OpenSPRTM instrument following the standard OpenSPRTM procedure. The test was run with PBS (pH7.4) at the maximum flow rate (150µL/min) to reach the signal baseline. A sample 200µL isopropanol run for 10 s to evacuate the air. After the baseline was reached, the PBS buffer was rinsed through the sample loop and evacuated with air. Slow down the flow rate of PBS (pH7.4) to 20µL/min, and then load 200µL of EDC/NHS (1:1) solution to activate the COOH sensor chip. 200µL of ligand E2 protein (0.4 mg/mL) was diluted for 4 min and the sample was rinsed with PBS (pH7.4). The sample with 200µL blocking solution and the sample loop was rinsed with PBS and evacuated with air. The baseline was observed for 5 min to ensure stability. Selected antibodies were diluted into a series of different concentrations and sampled at 20µL/min. Both antibodies and ligand binding times were 240 s and natural dissociation was 360 s. Kinetic parameters for the binding reactions were calculated using Trace Drawer software (Ridgeview Instruments AB), One to One analytical model.

### ELISA-based Mxra8-Fc binding assay

A fragment of cDNA encoding the Mouse-Mxra8 extracellular domain (residues 23–336, GenBank accession number NM_024263.4) or Human-Mxra8 extracellular domain (residues 24–337, GenBank accession number NM_032348.3) was appended with a TEV enzyme site and a human IgG1 Fc at the C-terminus as well as the IL-2 signal peptide at the N-terminus in pcDNA 3.4 expression vectors and transiently transfected into HEK293 cells followed by media collection and purification using protein A sepharose. The Mxra8-Fc binding assay was adapted from the previously described [27]. MaxiSorp ELISA plates (Corning) were coated with 2 µg/mL anti-mouse CHKV E2 monoclonal antibodies in CBS (pH9.6) overnight at 4 °C. The wells were washed four times with PBS and blocked with 4% BSA(Sigma-Aldrich) for 1 h at room temperature (RT). CHIKV virions (1 µg/ml) were diluted and added for 1 h at RT. After washing, MoNb-2E8 and MoNb-3C5 or Mouse Mxra8-Fc fusion protein (all at 10 µg/ml) were incubated for 30 min. Plates were washed and Human Mxra8-Fc (tenfold serial dilutions) were added to the plates and incubated for 1 h at RT. Plates were washed again and incubated with secondary rabbit anti-human IgG-Fc (1:5000, Bioss). After washing, the plates were developed with TMB substrate (TIANGEN) and 2 N H_2_SO_4_. Absorbance was measured at 450 nm.

### Indirect immunofluorescence assay

An indirect immunofluorescence assay was developed on CHIKV-infected Vero cells, as previously described [28]. Briefly, infected cells (multiplicity of infection (MOI), 1) were cultured for 72 h and then fixed in 4% paraformaldehyde (PFA) (Biosharp) for 20 min. PFA-fixed cells were permeabilized and blocked with 1% Triton X-100(MP)/1% BSA(Sigma-Aldrich)/PBS for 1 h at RT. The cells were incubated with antibodies (10 μg/mL) at 37 °C for 30 min, followed by Alexa Fluor 488 Anti-6 × His tag antibody (Abcam) or Cy3-labeled goat anti-rabbit IgG (H + L) (Beyotime) for 30 min at 37 °C. After washing with PBS, cells were observed under a fluorescence microscope (Mshot). Anti-CHIKV rabbit serum was obtained from a rabbit immunized with β-propiolactone CHIKV GD134 virion and used as a positive control. Anti-CHIKV E1 mouse IgG antibody (R&D Systems) was used as a control antibody.

### Flow cytometry assay

The binding of antibodies to the virus on the cell surface was assessed by flow cytometry assay. Monolayers of Vero cells were infected with CHIKV and ZIKA at an MOI of 0.1 and harvested at 48 h post-infection. Cells were then fixed with 4% paraformaldehyde, and permeabilized with permeabilization buffer (0.05% Triton-X in PBS). Cells were incubated on ice for 60 min with 10 μg/ml antibodies. After washing twice with PBS, cells were stained with 1:1000 diluted goat anti-human H&L (FITC) or FITC rabbit polyclonal to 6 × His tag (Abcam) for 45 min and analyzed using flow cytometry (Beckman CytoFlex, Brea, CA, USA).

### Human sera samples were detected in a sandwich ELISA

As secondary antibodies, bivalent VHHs were coupled with horseradish peroxidase (HRP) performed using a HRP Conjugation Kit (Abcam) following manufacturer instructions. 2 μg/mL of BiNb-2E8 in CBS (pH9.6) was added per well of a coated 96-well plate at 4 ℃ overnight. After washing with PBST and blocking with 5% BSA in PBST for 1 h, 100 μL of the E2 protein were diluted in a two-fold dilution series (starting dilution 5 in 10,240) or human sera samples (dilution 1:10) were added and incubated at RT for 1 h. Wells were washed 3 × 5 min, and 100μL of HRP labeled BiNb-3C5 (1 μg/mL) was added to each. After 1 h of incubation, plates were washed 5 times with PBST. TMB was added to the wells for colorimetric development and the absorbance was read at 450 nm. Human Mxra8-Fc served as the control. Samples were considered seropositive if *OD*_*450*_ values were higher than the mean obtained for the negative samples plus 3 standard deviations. The CHIKV positive sera samples were sourced from the Center for Disease Control and Prevention of Southern Theater Command. Twenty CHIKV negative sera from healthy subjects were used for the calculation and validation of the cut-off value and were excluded from the sample matrix.

### Epitope-binding using peptide-based ELISA

A pool of 10-mer peptides with 5 amino acid overlap spanning the Chikungunya/SL-CK1 E2 glycoprotein was generated by chemistry to a purity of 90% (GL Biochem). All peptides were provided as a lyophilized powder, reconstituted in dimethylsulfoxide (DMSO) to a concentration of 1 mg/mL, and stored at − 80 °C. Each peptide was coated at 1ug/mL in CBS (pH9.6) overnight at 4 °C and then blocked with 5% BSA(Sigma-Aldrich) in PBST. An irrelevant SARS-CoV-2 peptide was used as the negative control. The binding of the coated peptide was characterized by incubation with 2 µg/mL antibodies. Mouse monoclonal anti-6 × His tag HRP-conjugated (Abcam) was used at 1:5000 dilution in the blocking buffer, and further absorbance measurements of the enzymatic reaction in TMB substrate (TIANGEN), were used to detect the bound epitopes.

### Prediction of the conformation of VHH antibodies complex with CHIKV E2 protein

The homology model of protein structure was built on the DeepMind algorithm AlphaFold system (https://deepmind.com/). The complex model of Nb-2E8 or Nb-3C5 to the CHIKV E2 protein was generated by pyDOCK (https://life.bsc.es/pid/pydockweb). All structural data of the docking models were visualized using the PyMOL software (https://pymol.org/2/).

### Statistical analysis

All experiments were replicated at least two times, and representative data or pooled data from repeat experiments were recorded. Statistical analyses were carried out using GraphPad Prism Software (San Diego, CA, USA). Data were presented as the mean ± standard deviation (SD). Students t-test was used for two groups comparison. Two-sided *P*-values < 0.05 were considered statistically significant.

## Results

### Screening and identification of VHH antibodies targeting the E2 protein

**Fig. 1:**
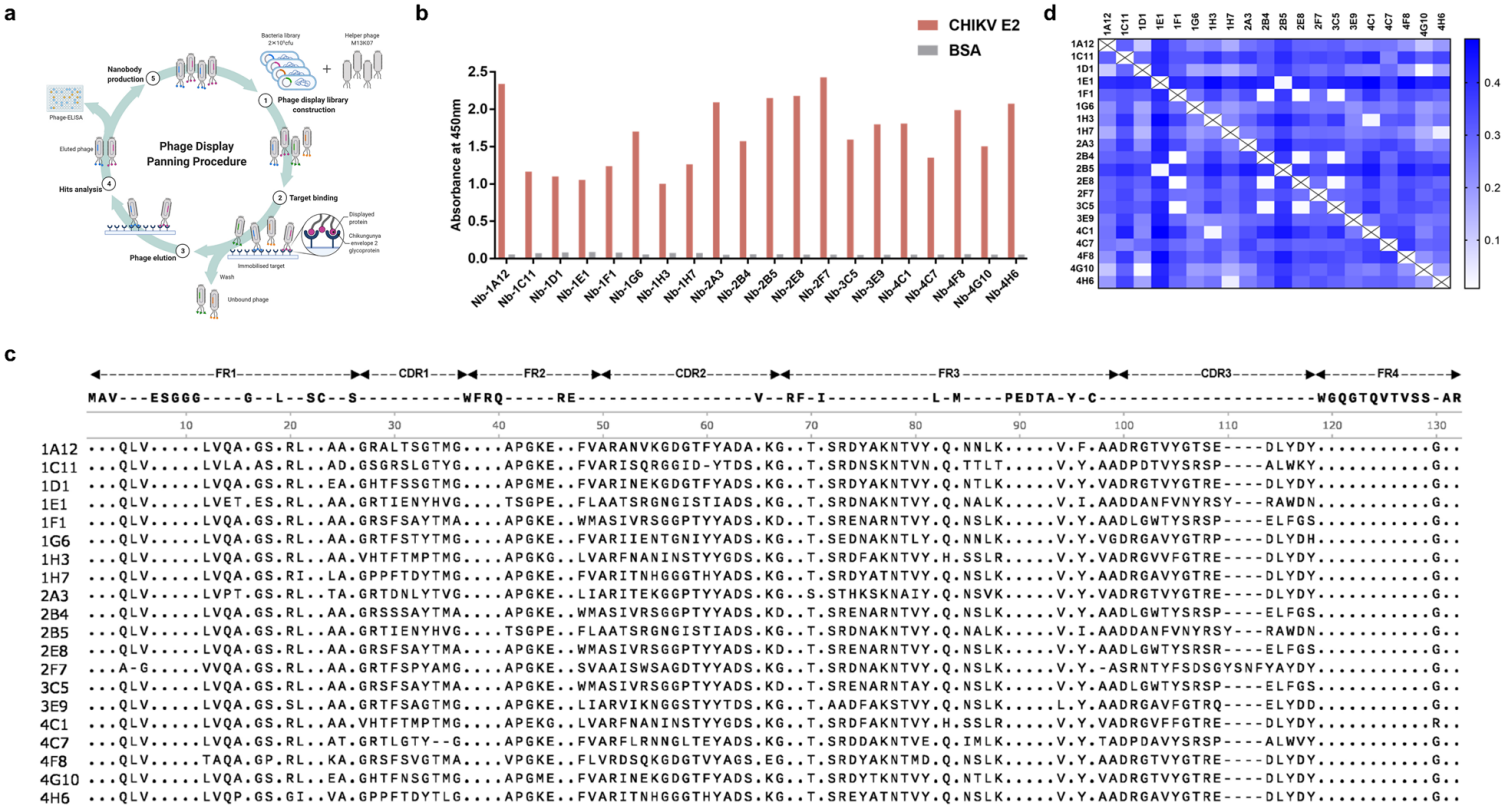
Screening the CHIKV E2 specific nanobodies from a VHH phage display library. **a** Biopanning procedure of candidate VHH domain. **b** Reactions from the 20 clones specifically binding with CHIKV E2 protein. **c** Alignment of the amino acid sequences of the isolated anti-CHIKV E2 nanobodies. **d** Overall similarity of VHH regions anti-CHIKV E2 nanobody sequences

VHH antibodies have become increasingly accessible using high-throughput naïve libraries to rapidly recognize multiple target antigens while resisting adverse biochemical properties such as instability, multi-reactivity, and aggregation during affinity maturation [29, 30]. To obtain active protein-binding clones from the enriched library, we conducted three rounds of screening (Fig. 1a) and examined for specific protein-binding using a phage ELISA. Additional file 2: Table S1 showed that the phage clones specific to the CHIKV E2 protein were effectively enriched through consecutive selection rounds. A total of 119 positive clones were included for genomic sequencing and data analyses (Additional file 2: Table S2). After CDRs sequence analysis, 20 unique CHIKV E2 specific antibodies were identified (Fig. 1b and 1c). These sequences were clustered into several discrete groups based on VHH similarities (Fig. 1d).

### Preparation and characterization of the VHH antibodies

**Fig. 2:**
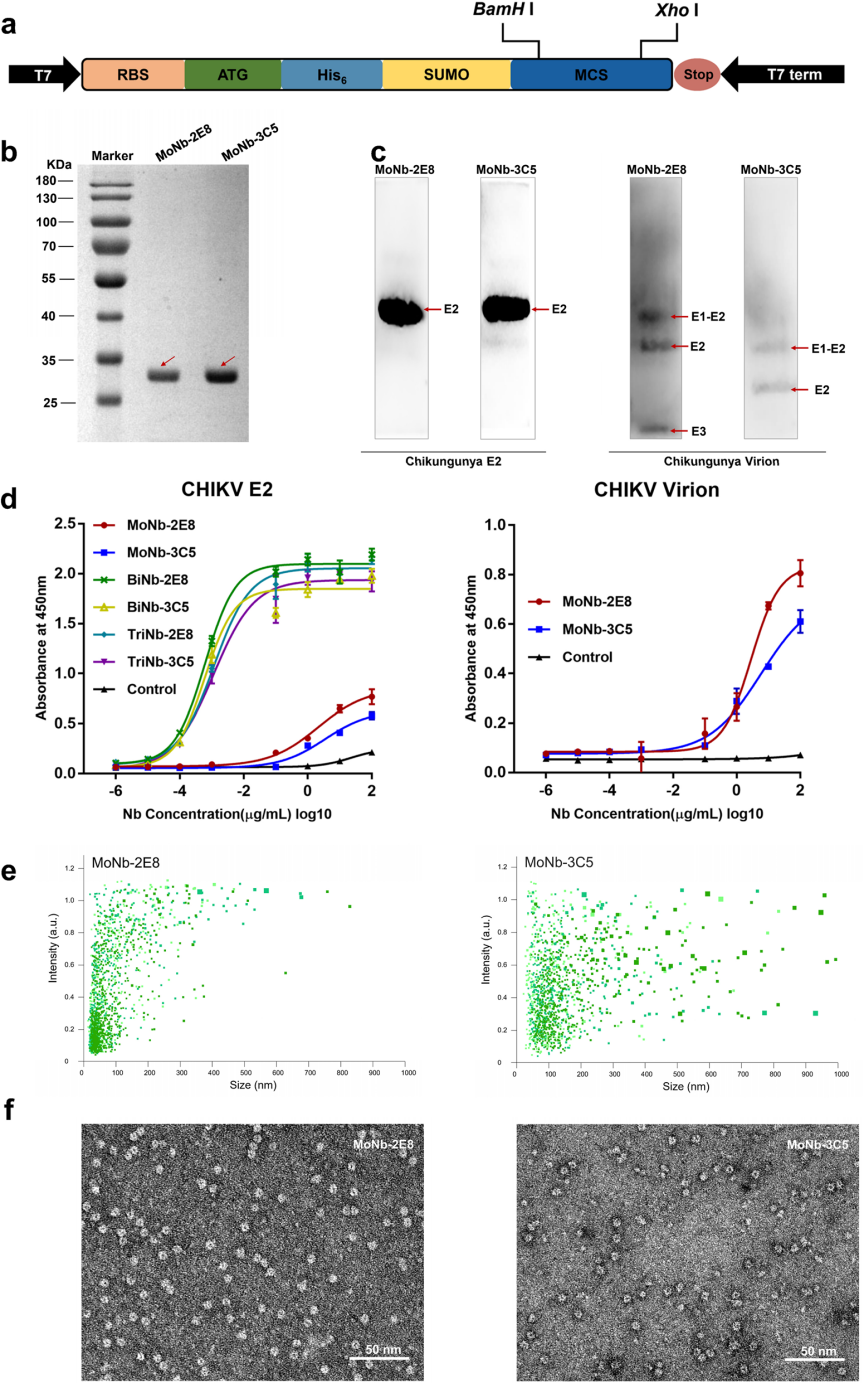
Fabrication, characterization and binding activity detection of nanobodies. **a** Schematic representation of the expression vector pET-SUMO for nanobody. **b** Soluble prokaryotic expression and purification of recombinant nanobody using an N-terminally 6 × His- and SUMO-tagged protein (~ 28 kDa).**c** Binding of SUMO-tagged VHHs to immobilized CHIKV E2 (~ 40kD) or Chikungunya virion was evaluated by western blot. **d** Binding of SUMO-tagged VHHs to immobilized CHIKV E2 or CHIKV virus was quantified by indirect ELISA. **e** Particle size distribution of SUMO-tagged VHHs by NTA. **f** Negative-stain TEM images of SUMO-tagged VHHs, Scale bar, 50 nm

Using the prokaryotic system in *E.coli* strain BL21(DE3), we expressed recombinant VHH antibodies with N-terminally 6 × His-and SUMO-tagged proteins (Fig. 2a). The expressed antibodies can exist as intracellular soluble and active proteins mostly found in the lysate supernatant (Additional file 1: Fig. S1). However, Nb-4C1 and Nb-4G10 were aggregated into inclusion bodies in the *E.coli* cytoplasm (Additional file 1: Fig. S1). Soluble VHHs were further purified by cobalt-chelating sepharose columns using 250 mM imidazole for elution. Coomassie-stained SDS-PAGE showed the recombinant proteins were detected at the expected apparent molecular weights, which was ~ 28 kDa (Fig. 2b). To confirm the specificity of binding of the 20 candidate VHH antibodies, CHIKV E2 as coating antigen was carried out by indirect ELISA (Additional file 1: Fig. S2). Of these, Nb-2E8 and Nb-3C5 were found to specifically bind with the E2 protein as well as CHIKV virion (Fig. 2d). Western blot analysis showed that both Nb-2E8 and Nb-3C5 were able to recognize the purified CHIKV virion and the E2 protein (Fig. 2c). The results suggested that both antibodies recognize linear epitopes on CHIKV E2. Moreover, NTA analysis revealed the particles in the size range characteristic for Nb-2E8 and Nb-3C5 (< 100 nm) (Fig. 2e). The morphology of VHHs was further confirmed by TEM (Fig. 2f).

### Binding and identification profiles for MoNb-2E8 and MoNb-3C5

**Fig. 3:**
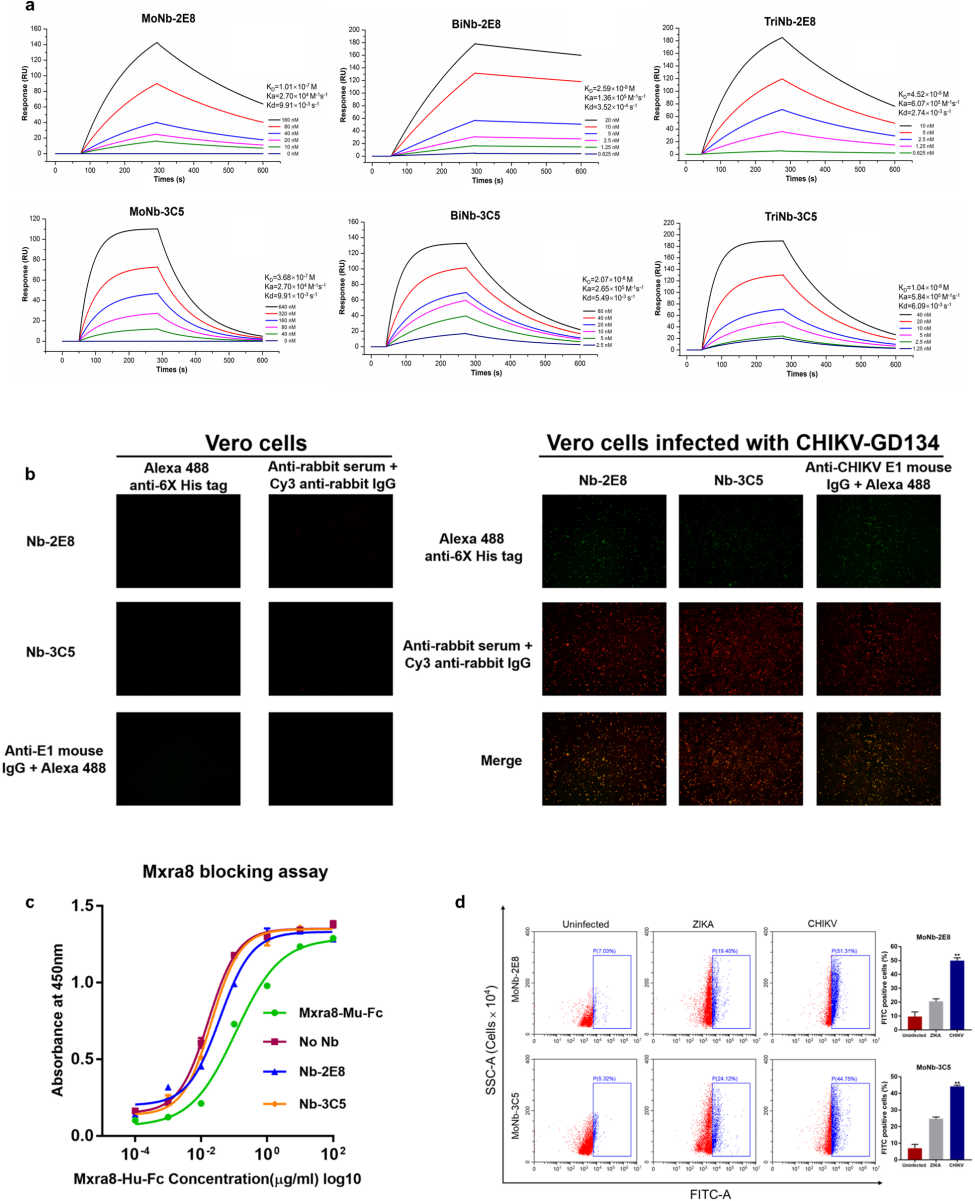
Biophysics of the nanobodies binding to CHIKV E2. **a** Binding kinetic for nanobodies were obtained by LSPR. **b** CHIKV E2 protein expression in infected cells was analyzed by immunofluorescence staining using Nb-2E8 and Nb-3C5. **c** The abilities of Nb-2E8 and Nb-3C5 to block CHIKV E2 interaction with Mxra8 were determined by a competition ELISA. **d** Cell binding of the nanobodies were quantified by flow cytometry. Left: representative flow cytometry plots; right: bars show mean ± S.E.M

To validate the binding capabilities of the antibodies targeting the E2 protein, we measured the binding kinetics of antibodies by LSPR. While MoNb-2E8 and MoNb-3C5 have an affinity of 101 nM and 368 nM (Fig. 3a), they showed a weak affinity with the E2 protein. We also evaluated whether Nb-2E8 and Nb-3C5 compete with Mxra8-Fc to bind with CHIKV. Both Human Mxra8-Fc and Mouse Mxra8-Fc could non-competitively bind to CHIKV (Fig. 3c). This would explain that Nb-2E8, Nb-3C5, and Mxra8 interact at different sites with virions and most likely do not compete for the same epitope. Nb-2E8, Nb-3C5, and anti-rabbit serum were used as primary antibodies to detect the expression of the viral E2 protein in Vero cells at 72 h post-infection. Figure 3b showed that specific green and red fluorescence were observed in the virus-infected cells, but not in virus-uninfected cells. Further confirmation of the binding specificity of the VHHs was accomplished by flow cytometry analysis of infected cells labeled with Nb-2E8 and Nb-3C5 (Fig. 3d). Blue boxes, the proportion of cells in which the fluorescence value exceeded the gating threshold. Quantifications presented in ratios of fluorescence-positive cells to the total number of cells, revealed that the FITC-positive values of CHIKV infected cells were visibly higher than ZIKA infected cells (*P* < 0.05).

### Establishment of the sandwich ELISA

**Fig. 4:**
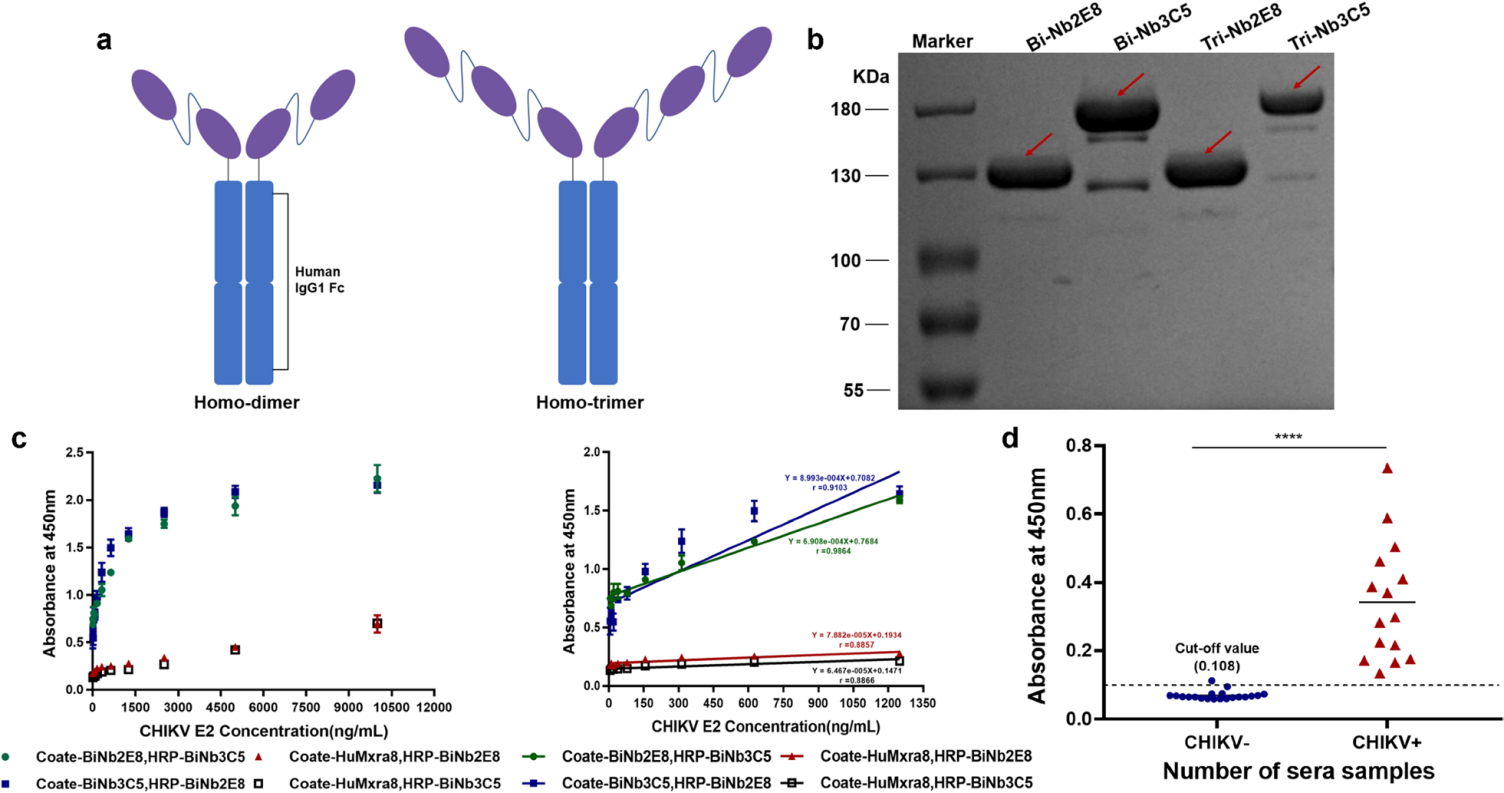
Features and applications of multivalent nanobodies. **a** A schematic of multivalent nanobodies. **b** SDS-PAGE of multivalent nanobodies under non-reducing condition. **c** Establishment of standard curve using HRP-labeled bivalent nanobodies. **d** Validation of the sandwich ELISA using CHIKV-positive serum samples

To further improve the affinity of VHH antibodies against CHIKV E2, Nb-2E8 and Nb-3C5 were fused to an Fc domain of IgG1 to generate multivalent fusion proteins and obtain milligram quantities of highly pure (> 95%) recombinant proteins (Fig. 4a, 4b, and Additional file 2: Table S3). BiNb-2E8, BiNb-3C5, TriNb-2E8, and TriNb-3C5 showed high binding affinities to CHIKV E2 with equilibrium dissociation constants (KD) of 2.59, 20.70, 4.52, and 10.40, respectively (Fig. 3a). Cytofluorimetric quantification of BiNb-2E8 stained cells to the total number of cells was 95.81% and 95.20% for BiNb-3C5 (Additional file 1: Fig. S3). We developed a sandwich ELISA protocol, in which purified bivalent antibodies are used as the capture and different bivalent antibodies are used for detection, to quantify relative antigen response levels. From 4 independent experiments, the linear equation of Y = 6.908e-004 X + 0.7684 (*r* = 0.9864, *P* < 0.0001) was found to be the optimal linear fit, which demonstrated good linearity over the concentration range (5–1250 ng/mL) (Fig. 4c). The BiNb-2E8 was combined as capture and the HRP-labelled BiNb-3C5 as detection to reveal a combination with good performance.

### Validation with human sera samples

To establish a baseline for the evaluation of the E2 antigen capture test, 20 CHIKV seronegative samples were analyzed. The cut-off was defined as the mean *OD*_*450*_ value of the negative controls + 3SD: 0.0696 + (3 × 0.0128) = 0.108. Hence, sera samples giving *OD*_*450*_ values higher than 0.108 were recorded as positive, and negative when *OD*_*450*_ values were lower than 0.108. By this criterion, all 15 serum of patients infected with CHIKV had OD values greater than that of the cut-off value, which was judged as positive. Also, the difference between CHIKV seronegative and seropositive samples was significant s (*P* < 0.0001) (Fig. 4d). Therefore, the sandwich ELISA established in this study can be used in the early diagnosis of CHIKV viral infection.

### Mapping linear epitopes of the E2 protein using Nb-2E8 and Nb-3C5

**Fig. 5:**
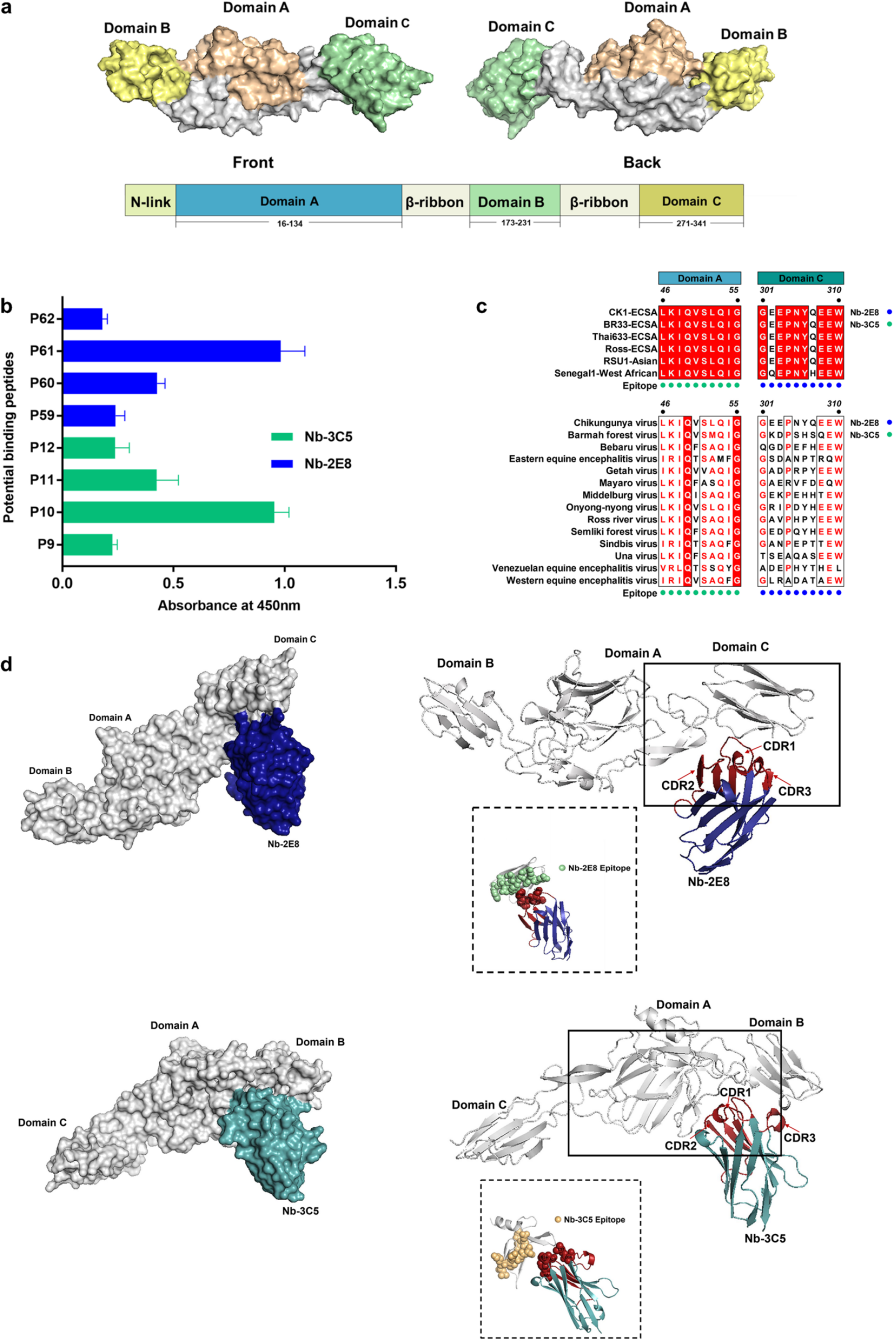
Distribution profiles of components of E2-Nb complexes and their corresponding epitopes. **a** Schematic diagram of domains and positions of the CHIKV E2 Glycoprotein. **b** Potential binding peptides response to the Nb-2E8 and Nb-3C5 as measured by a peptide-ELISA. **c** Alignment of the Nb-2E8 and Nb-3C5 epitope region with E2 sequences of different genotype strains and alphaviruses. **d** Structure docking model of Nb-2E8 and Nb-3C5 bound to CHIKV E2 protein

To characterize specific linear epitopes of the CHIKV E2 protein, 68 peptides with an offset of ten amino acids were used to assess responses (Additional file 2: Table S4). As shown in Fig. 5b, P61- GEEPNYQEEW and P10-LKIQVSLQIG strongly reacted with Nb-2E8 and Nb-3C5, which correspond to the amino acid residues 331 to 340 and 46 to 55 of CHIKV E2 domain C and A, respectively (Fig. 5a). Comparison of epitopes of the Nb-2E8 and Nb-3C5 with sequences from representative strains of each CHIKV genotype (SL-CK1, BR33, CU-Chik683, and Ross: East, Central, and South African (ECSA); RSU1: Asian; Senegal: West African) (Fig. 5c). Of note is that the Nb-3C5 recognized epitope is highly conserved across the six-representative CHIKV genotypes. And two amino acids at sites were fairly well conserved among other alphaviruses genera. The Nb-2E8 epitope had two residue variations at positions 302 (E-Q) and 307 (Q-H) in the West African genotype of CHIKV and was not identical among all representative alphaviruses. For visualization, a computer docking simulation was used to map the binding of antibodies to the E2 protein. Like peptide-based ELISA, the formation of complex demonstrates Nb-2E8 and Nb-3C5 bind different epitopes on E2 (Fig. 5d). In the complex**,** residues L98, G99, W100, T101, and L108 from the CDR3 loop of Nb-2E8 made contacts with the P61epitopes of E2. The CDR1 and CDR3 regions of Nb-3C5 have contributed to the P10 epitopes of domain C.

## Discussion

Nanobody opens important possibilities for biomedical applications, but so far, most have been generated by immunized Camelidae, which is time-consuming and expensive for laboratories. Naïve phage display library, a technique for obtaining genetically engineered nanobodies against an antigen without immunization [31], allowed the isolation of antibodies against autoantigens, non-immunogenic, or toxic antigens. High-affinity antibodies can be obtained in a short time when the library capacity is sufficiently large. VHH, also namely nanobody, is tenfold smaller than conventional antibodies with unique advantages [32]. As a result of its small size and stability, it is easily incorporated into the body’s natural metabolic pathways. More importantly, VHH domains have longer complementary antigen-binding regions CDR3, providing greater antigen-binding capacity, and the affinity of nanobodies can be further improved by genetic engineering techniques [33]. In this study, we performed genetically engineered VHH antibodies targeting of CHIKV by using a phage display technique based on a naïve antibody library derived from 103 healthy adult alpaca lymphocytes with a capacity of 2 × 10^9^ cfu, which was able to screen for specific binding VHH antibodies. A total of 20 VHH sequences specifically binding to CHIKV E2 were obtained, including CDR1, CDR2, and CDR3. The amino acid sequence length of the CDR3 region varies from 17 to 20, with 85% of the sequences greater than 17 amino acids, indicating typical characteristics of a nanobody. In framework region 2 (FR2), the high stability and solubility of VHH was attributed to the typical substitutions of hydrophobic residues. Altogether, the VHH antibody was a convenient monovalent scaffold, with the single-chain easily expressed in *E. coli*.

The pET-SUMO expression system, which combines the pET plasmid with a SUMO partner linking the target gene to SUMO via a homologous recombination cloning strategy, is efficiently and stably expressed under the control of a strong T7 promoter [34]. The SUMO protein was an ideal candidate tag for enhancing the expression, folding, and stability of proteins [35, 36], especially for heterologous proteins like antibody fragments [37]. Several studies have reported that recombinant proteins have been efficiently produced using the SUMO fusing system in *E. coli* [38–40]. The results from this study agree with the previous studies. VHH antibodies were secreted to the culture medium in a soluble form. An N-terminal 6 × His Tag was added to the expression vector to aid purification and, SDS-PAGE analysis revealed a single band at approximately 28 kDa. However, two VHHs Nb-4C1 and Nb-4G10 were expressed in *E. coli* and refolded from inclusion bodies. We speculated that it can potentially be associated with VHH sequences.

For purified VHH antibodies, following identification by ELISA, western blot, and IFA-mediated overlap experiment, Nb-2E8, and Nb-3C5, we have chosen for further validation. SPR technology is widely known as a golden standard for capturing antibody-antigen interactions [41, 42]. To determine the kinetic rate and affinity constants, binding analysis of E2 and monovalent antibodies were carried out by LSPR. LSPR results demonstrated that the binding affinity of MoNb-2E8 and MoNb-3C5 is relatively low. Recombinant fusions of monovalent antibodies are limited by weak binding affinity due to the lack of multivalent affinity benefits, poor production capacity, and potential immunogenicity [43–45]. To further improve the affinity of VHHs against CHIKV E2, homo-dimer (BiNb-2E8, BiNb-3C5) and homo-trimer (TriNb-2E8, TriNb-3C5) were fused to an Fc domain of IgG1 to generate multivalent fusion proteins. We surprisingly found that compared with the corresponding monovalent antibodies, both homo-dimer and homo-trimer showed a 100-fold enhancement towards the E2 protein. Among them, BiNb-2E8 was confirmed by LSPR analysis, with a binding affinity of ~ 2.59 nM. Developing high affinity and specific antibodies for capturing viral antigens is crucial for any point-of-care testing (POCT) such as ELISA to be successful. Thus, we capitalize on the strengths of the selected VHH antibodies to establish a rapid, convenient, and reliable screening test.

A major advantage of the ELISA method is that it can detect viruses at a concentration of 1–10 µg/ml and require few antibodies [46]. Traditional antibodies considered for developing ELISA have been polyclonal and monoclonal antibodies. However, these highly specific antibodies are cost-effective and have poor stability under adverse environmental conditions [47]. Nanobodies are powerful tools for a wide application in molecular biology, providing high affinity and antigen specificity. In this study, we prepare specific multivalent antibodies against the E2 protein and develop a double-antibody sandwich ELISA for the detection of antigen quality using BiNb-2E8 as capture antibodies and BiNb-3C5 as the detection antibodies. A good linear correlation was achieved in the 5–1000 ng/mL range. ELISA results showed that patients with CHIKV had significantly higher serum E2 levels than CHIKV seronegative (*P* < 0.0001). Furthermore, the best choice for coated antibodies is to recognize only a single antigenic determinant [48–50]. Two types of diagnostic and protective antigens, ZIKA and SARS-CoV-2, were tested by indirect ELISA. Nb-2E8 and Nb-3C5 were found to have no binding activity to react with the  ZIKA and SARS-CoV-2 proteins without any dose-dependent manner. Additionally, flow cytometry analysis of the two VHHs of Nb-2E8 and Nb-3C5 positively stains CHIKV-infected cells but was negative on ZIKA-infected cells. Overall, Nb-2E8 and Nb-3C5 were specific for CHIKV and had no cross-reactivity with ZIKA and SARS-CoV-2. These findings indicated that the ELISA protocol could be performed in any laboratory and served as the foundation of an inexpensive tool for the early diagnosis of CHIKV.

An understanding of the interaction between the antibody and its targeted antigen, and knowing the epitopes, are critical for developing epitope-based diagnostic reagents. The epitopes recognized by the VHHs were identified by peptide-based ELISA, and the epitopes sites were located. Previous studies have reported that several monoclonal antibodies targeting the structure of the domain A and B could block fusion and inhibit interaction with cellular receptors [8, 51]. Epitope mapping revealed that Nb-2E8 and Nb-3C5 bound the different linear epitopes distributed in domain C and domain A, respectively. In particular, the P10-^46^ “LKIQVSLQIG”^55^ has not been reported. But we had previously predicted that the peptide “QVSLQIGIK” was the immunodominant B- and T-cell epitope using in *silico* techniques (unpublished data). Several studies suggested that the P61-^301^ “GEEPNYQEEW”^310^ was found to be B-cell epitopes shared by humans and mice [52, 53]. Unfortunately, the selected two VHH antibodies that focus on the domains of the target linear epitopes are non-neutralizing. The main neutralization sites on the CHIKV E2 protein are likely to be available in a conformation-dependent manner, rather than as linear epitopes.

Based on sequence alignment analysis, the P10 was completely conserved among all CHIKV genotype strains analyzed here, indicating that Nb-3C5 is potentially capable of recognizing three CHIKV genotypes (ECSA, Asian, and West African). It also found that the P61 epitope sequence in the West African lineage CHIKV strain had two single two amino acid differences from the other genotypes. This may limit the potential diagnostic applications of Nb-2E8 in countries where West African lineage strains of the virus circulate. However, all epitopes had significant amino acid mutations across other alphaviruses (Mayaro virus, O’nyong’nyong virus, Semliki Forest virus, Ross River virus, Sindbis virus, etc.). These features enable the differential diagnosis of CHIKV. Taken together, these VHH antibodies, Nb-2E8 and Nb-3C5, specifically recognize different epitopes of the CHIKV E2 protein, which are the optimal pair of antibodies for the development of a double-antibody sandwich ELISA.

There are several potential limitations to the study. First, our study is only focused on highly potent antibodies against CHIKV. Further studies are needed to demonstrate other VHH antibodies could interact with the E2 protein and to evaluate the diagnostic or treatment merit of specific antibodies. Second, the present study illustrates a strategy to mine the valuable VHH antibodies by naïve phage display library. The results support the feasibility of VHHs as a rapid diagnostic tool. However, the diagnostic accuracy parameters and procedures in this study have not been directly optimized. To establish a standardized CHIKV diagnostic process, the optimization of antibody reagents is required. Third, the binding site prediction of nanobody-E2 complex relies on peptide-ELISA and homology-modeling applications, which lacked X-ray diffraction (XRD) or cryoelectronic microscopy to investigate the crystal structure parameters.

## Conclusions

In summary, we developed VHH antibodies from a naïve phage display library that addresses the specific needs of CHIKV serodiagnosis. The selected antibodies of Nb-2E8 and Nb-3C5 could bind the CHIKV E2 protein with high specificity and affinity in a multivalent platform. These results suggest that the selected antibodies are used for diagnostic applications to detect sera binding protein and/or virions, either as single VHHs or in combination.

## Supplementary Information

**Additional file 1: Fig. S1**. Expression of 20 candidate nanobodies using the *E.coli* system. **Fig. S2**. Reactivities of 18 to the E2 protein of CHIKV measured by indirect ELISA. **Fig. S3**. Representative flow cytometry plots and data analysis of mean fluorescence value. A representative experiment among 2 is shown.

**Additional file 2: Table S1**. 119 binders were screened using a phage ELISA. **Table S2**. 119 binders were screened using a phage ELISA. **Table S3**. Properties and expression of multi-valent nanobodies. **Table S4**. Overlapped peptides covering the CHIKV E2 protein in this study.

## Data Availability

All the data analyzed throughout this study are included in the article.
