# Supplementary material for: Highly potent multivalent VHH antibodies against Chikungunya isolated from an alpaca naïve phage display library
Source: J Nanobiotechnology. 2022 May 14;20:231. doi: 10.1186/s12951-022-01417-6 (PMC9107221; doi:10.1186/s12951-022-01417-6)
Supplement: Supplementary file 1 — Additional file 1: Fig. S1. Expression of 20 candidate nanobodies using the E.coli system. Fig. S2. Reactivities of 18 to the E2 protein of CHIKV measured by indirect ELISA. Fig. S3. Representative flow cytometry plots and data analysis of mean fluorescence value. A representative experiment among 2 is shown. [file 12951_2022_1417_MOESM1_ESM.docx]

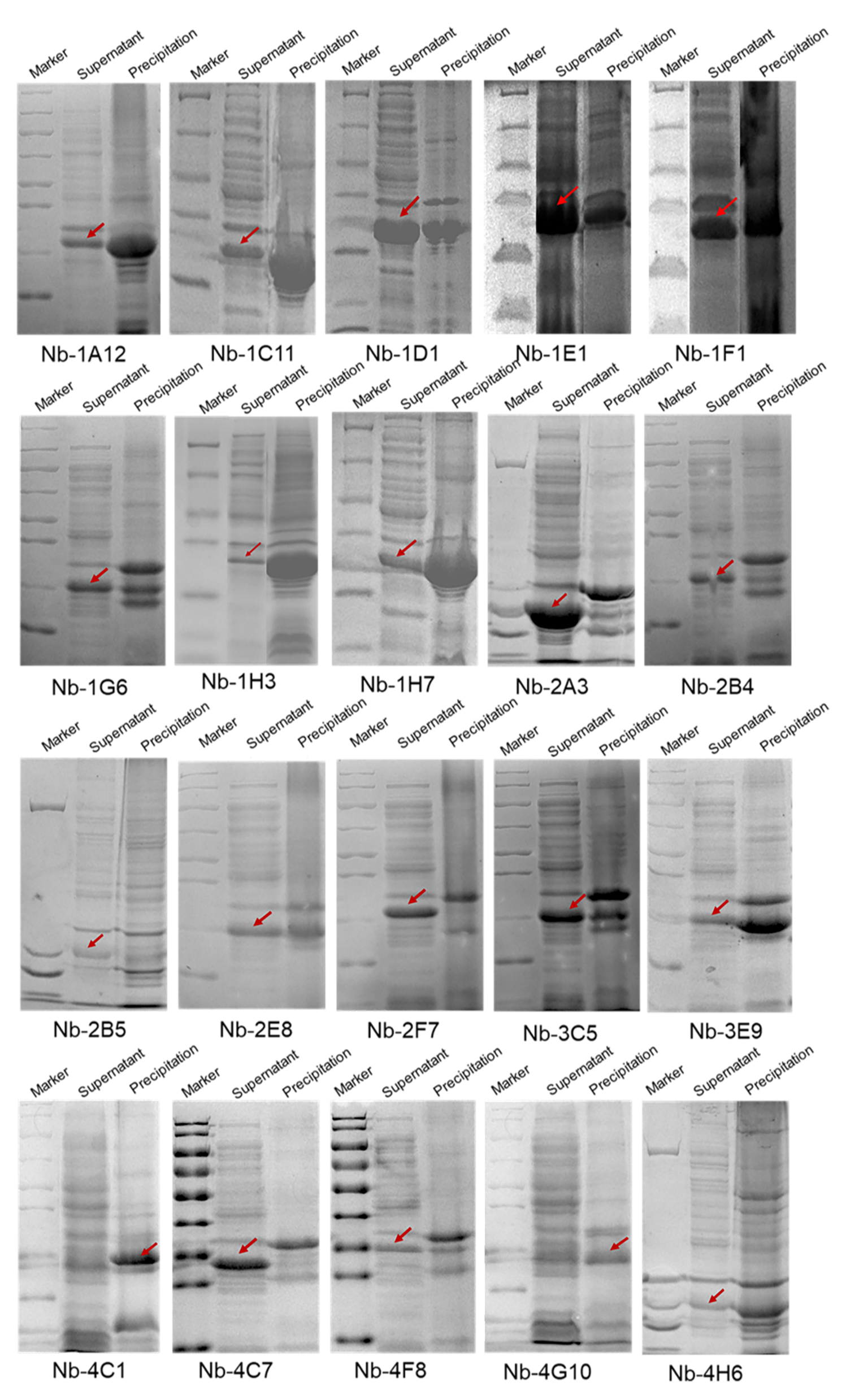
**Fig S1.** Expression of 20 candidate nanobodies using the *E.coli* system.


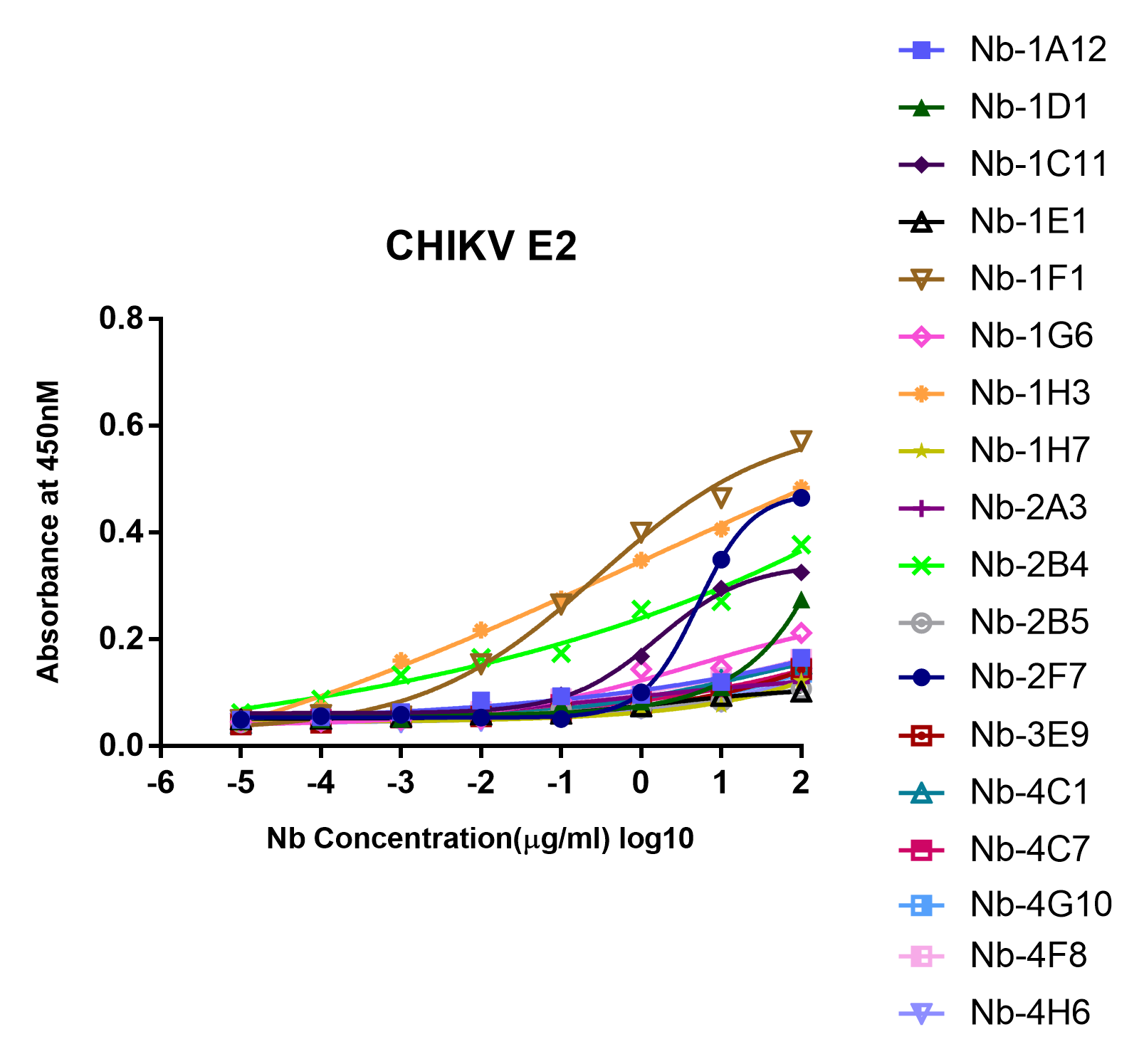
**Fig S2.** Reactivities of 18 to the E2 protein of CHIKV measured by indirect ELISA.


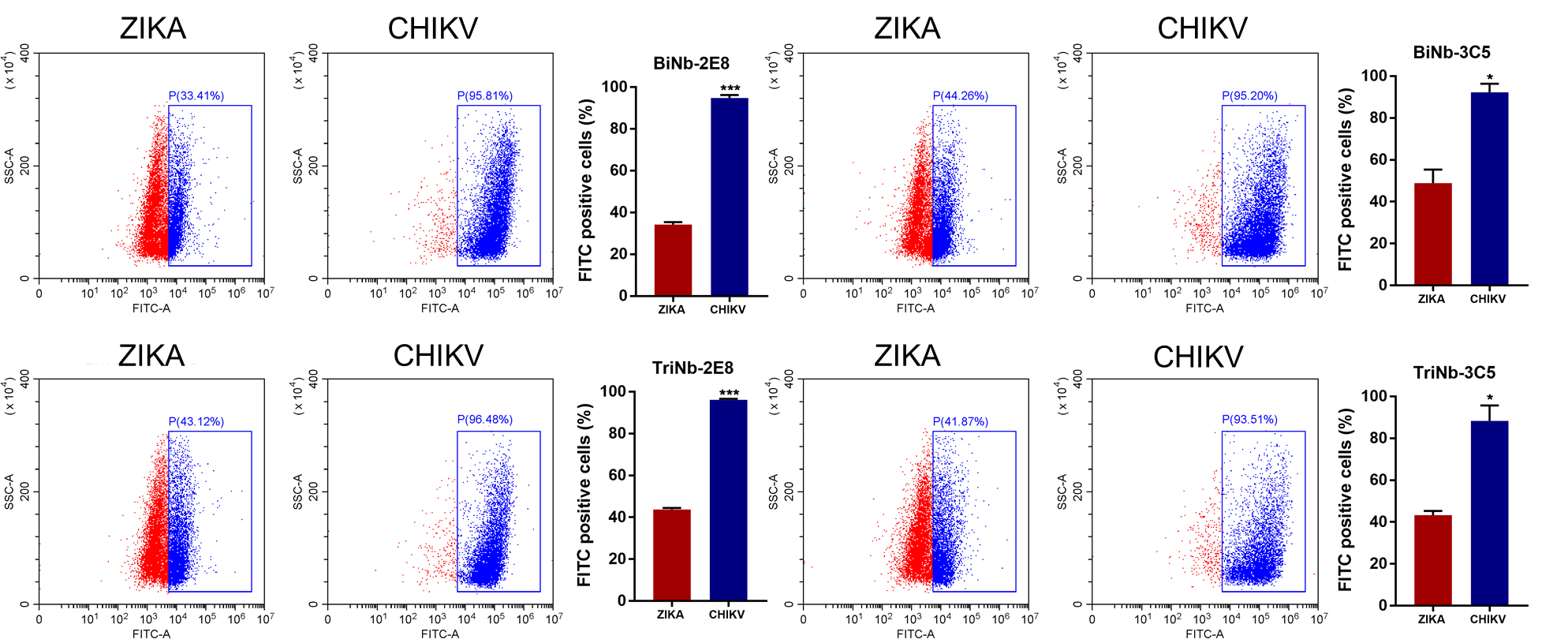
**Fig S3.** Representative flow cytometry plots and data analysis of mean fluorescence value. A representative experiment among 2 is shown.
